# Mechanisms by Which Licochalcone E Exhibits Potent Anti-Inflammatory Properties: Studies with Phorbol Ester-Treated Mouse Skin and Lipopolysaccharide-Stimulated Murine Macrophages

**DOI:** 10.3390/ijms140610926

**Published:** 2013-05-24

**Authors:** Han Na Lee, Han Jin Cho, Do Young Lim, Young-Hee Kang, Ki Won Lee, Jung Han Yoon Park

**Affiliations:** 1 Department of Food Science and Nutrition, Hallym University, Chuncheon 200-702, Korea; E-Mails: 20063716@hallym.ac.kr (H.N.L.); hanjini@hallym.ac.kr (H.J.C.); ldydo@hanmail.net (D.Y.L.); yhkang@hallym.ac.kr (Y.-H.K.); 2 WCU Biomodulation Major, Department of Agricultural Biotechnology and Center for Food and Bioconvergence, Seoul National University, Seoul 151-921, Korea; E-Mail: kiwon@snu.ac.kr; 3 Advanced Institutes of Convergence Technology, Seoul National University, Suwon, Gyonggi-do 443-270, Korea

**Keywords:** licochalcone E, inflammation, mouse skin

## Abstract

In this study we found that licochalcone E (LicE), a recently isolated retrochalcone from *Glycyrrhiza inflata*, exhibits potent anti-inflammatory effects in 12-*O*-tetradecanoylphorbol-13-acetate (TPA)-induced mouse ear edema and lipopolysaccharide (LPS)-stimulated RAW 264.7 murine macrophage models. Topical application of LicE (0.5–2 mg) effectively inhibited TPA-induced (1) ear edema formation; (2) phosphorylation of stress-activated protein kinase/c-Jun-*N*-terminal kinase (SAPK/JNK), c-Jun, and extracellular signal regulated kinase 1/2; and (3) expression of inducible nitric oxide synthase (iNOS) and cyclooxygenase (COX)-2 proteins in mouse skin. The treatment of RAW 264.7 cells with LicE (2.5–7.5 μmol/L) induced a profound reduction in LPS-induced (1) release of NO and prostaglandin E_2_; (2) mRNA expression and secretion of interleukin (IL)-6, IL-1β and tumor necrosis factor-α; (3) promoter activity of *iNOS* and *COX-2* and expression of their corresponding mRNAs and proteins; (4) activation of AKT, p38 mitogen activated protein kinase (MAPK), SAPK/JNK and c-Jun; (5) phosphorylation of inhibitor of κB (IκB) kinase-αβ and IκBα, degradation of IκBα, translocation of p65 (RelA) to the nucleus and transcriptional activity of nuclear factor (NF)-κB; and (6) transcriptional activity of activator protein (AP)-1. These results indicate that the LicE inhibition of NF-κB and AP-1 transcriptional activity through the inhibition of AKT and MAPK activation contributes to decreases in the expression of pro-inflammatory cytokines and the inducible enzymes iNOS and COX-2.

## 1. Introduction

Inflammation performs an important role as the first line of host defense mechanisms against infection of pathogenic microorganisms and functions to clear injurious materials (reviewed in [1–3]). However, over the past decade, it has become widely accepted that improper resolution of inflammatory networks is centrally involved in the pathogenesis of many acute and chronic diseases. Thus, inflammation is a driving force behind a wide variety of chronic diseases including cancer, diabetes, obesity and Alzheimer’s disease [4]. Therefore, the down-regulation of inflammation-related risk factors could be an excellent strategy to prevent or delay these chronic diseases.

Macrophages are the primary pro-inflammatory cells and the activation of macrophages plays a vital role in the initiation and propagation of inflammatory responses by production of cytokines including interleukin (IL)-6, IL-1β and tumor necrosis factor (TNF)-α, nitric oxide (NO), prostaglandin E_2_ (PGE_2_) and other inflammatory mediators (reviewed in [5,6]). NO is a diatomic free radical molecule synthesized from L-arginine in a reaction catalyzed by a family of enzymes known as NO synthases (NOSs). There are three isoforms of NOS: neuronal NOS, endothelial NOS, and inducible NOS (iNOS) [7]. Inflammatory signals, such as bacterial products (e.g., lipopolysaccharide (LPS), lipoteichoic acid, DNA, RNA) or proinflammatory cytokines (e.g., IFN-γ, TNF-α, IL-1), induce the expression of iNOS [8]. Induction of iNOS leads to overproduction of NO, which may cause tissue damage and toxicity leading to significant immunopathological conditions in the host [9]. Cyclooxygenase (COX)-2 is another enzyme that plays a pivotal role in the mediation of inflammation by catalyzing the rate-limiting step in PG biosynthesis. In inflammatory and malignant conditions, pro-inflammatory cytokines (IL-1β, TNF-α and epidermal growth factor) and mutagenic substances induce the expression of COX-2 [10,11].

The main signal transduction molecules in macrophages that mediate inflammatory responses include proteins in the nuclear factor-kappaB (NF-κB) and mitogen-activated protein kinase (MAPK) families (reviewed in [12,13]). NF-κB is a transcription factor formed by the dimerization of proteins in the Rel family. It is bound to the inhibitors of κB (IκB) in unstimulated cells [14]. NF-κB regulates the expression of a wide variety of genes that encode inflammatory cytokines, chemokines, inducible enzymes such as COX-2 and iNOS, adhesion molecules and growth factors (reviewed in [13]). MAPKs phosphorylate various cytoplasmic substrates. One of the major downstream target molecules for MAPKs is activator protein (AP)-1 [15]. The transcription factor AP-1 also regulates the expression of these inflammatory mediators [16–18].

Identification of bioactive natural compounds with anti-inflammatory properties and investigation of their anti-inflammatory mechanisms can be an effective strategy to reduce the incidence and severity of inflammation, thereby reducing the risk of a wide variety of diseases related to inflammation. Licorice has been used not only in foods such as sweeteners but also as medicine for thousands of years. In Oriental traditional medicine, licorice has been used in the treatment of a wide variety of diseases ranging from peptic ulcers to tuberculosis [19]. Licochalcones are the major phenolic constituents of the licorice species *Glycyrrhiza inflata* that has been shown to exert anti-inflammatory properties [20]. Licochalcone A has been reported to have anti-inflammatory and anti-cancer effects [21,22].

**Figure 1:**
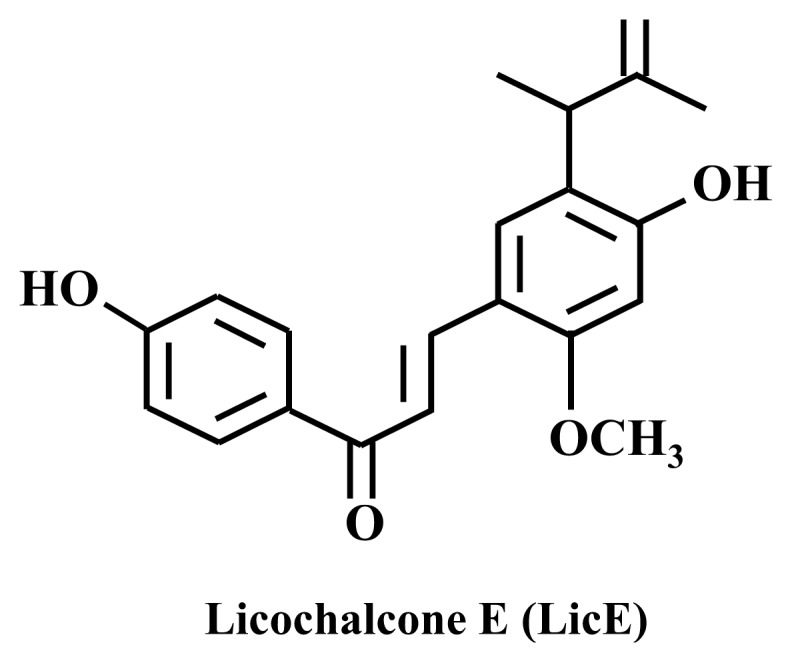
Chemical structure of licochalcone E (LicE).

Licochalcone E (LicE, Figure 1) was recently isolated and characterized from the roots of *Glycyrrhiza inflata* [23]. In the present study, we examined whether LicE exhibits anti-inflammatory properties in mice using the 12-*O*-tetradecanoylphorbol-13-acetate (TPA)-induced ear edema model and investigated the underlying mechanisms in detail using RAW 264.7 cells.

## 2. Results and Discussion

### 2.1. LicE Suppresses TPA-Induced Ear Edema in Mice

**Figure 2:**
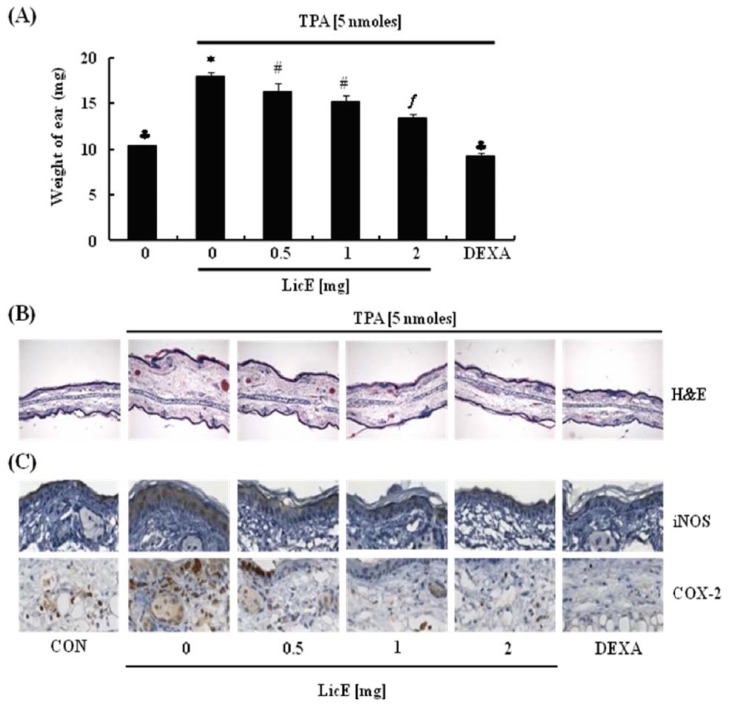

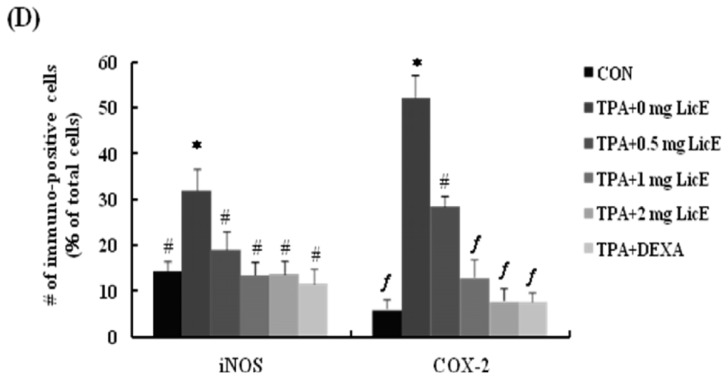
Licochalcone E (LicE) inhibits TPA-induced ear edema formation in mice. LicE (0, 0.5, 1, or 2 mg) was topically applied to the mouse ear 1 h prior to the topical application of 5 nmoles of TPA (4 h). (**A**) The weights of 6 mm-diameter ear punch samples were measured. Each bar represents the mean ± SEM (*n* = 10); (**B**) Ear sections were stained with hematoxylin and eosin (H&E). Representative images of H&E stained ear sections are shown; (**C**) Ear sections were stained with an antibody raised against iNOS or COX-2 and counterstained with hematoxylin. Representative images of the immunohistochemical analysis are shown; (**D**) The total number of hematoxylin-stained nucleus/field was counted and set at 100%. The number of iNOS- or COX-2-positive cells were expressed as a percentage of the total number of cells. Each bar represents the mean ± SEM (*n* = 3). Means without a common symbol are statistically different (*p* < 0.05). DEXA, dexamethasone.

To determine whether LicE exerts anti-inflammatory properties in animals, LicE or vehicle was topically applied to ears of ICR mice one hour prior to TPA treatment. The degree of inflammation was estimated by measuring the weight of ear tissue obtained by ear punch. TPA treatment increased the ear weight and LicE treatment dose-dependently reduced the TPA-induced increase in ear weight (Figure 2A). The thickness of the ear was examined histologically after H&E staining. LicE suppressed TPA-induced increases in ear thickness (Figure 2B). The average ear thickness was quantified from H&E-stained ear sections. Ear thickness (μm) in control, TPA, TPA + 0.5 mg LicE, TPA + 1 mg LicE, TPA + 2 mg LicE, and TPA + dexamethasone group was 289 ± 18, 587 ± 16, 470 ± 28, 414 ± 28, 374 ± 38, and 290 ± 7, respectively. IHC analysis of the pro-inflammatory enzymes iNOS and COX-2 proteins revealed that TPA-induced increases in the expression of iNOS and COX-2 proteins were significantly suppressed by LicE (Figure 2C,D). Dexamethasone was very effective in inhibiting ear edema formation as well as the expression of iNOS and COX-2 (Figure 2A–D).

### 2.2. LicE Suppresses LPS-Induced mRNA Expression of Inflammatory Mediators and Enzymes, as well as iNOS and COX-2 Promoter Activities in RAW 264.7 Cells

**Figure 3:**
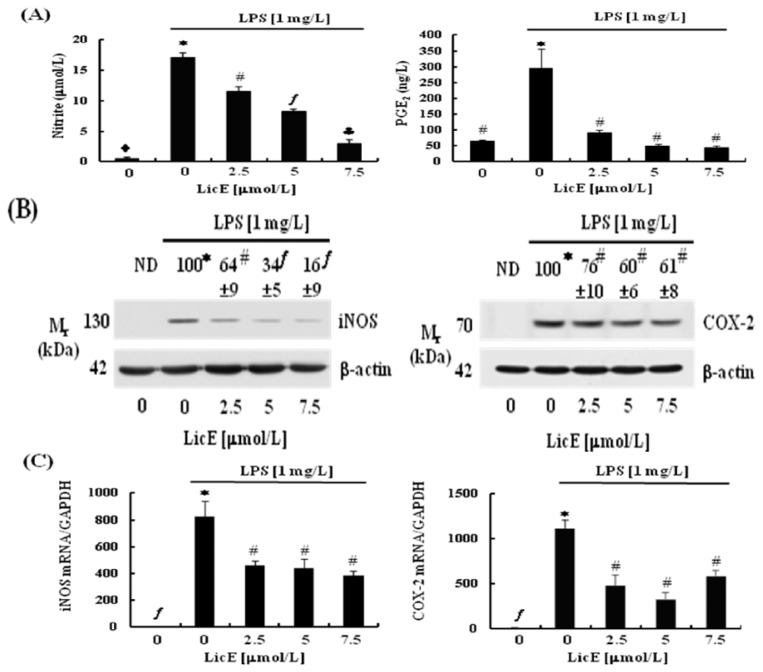

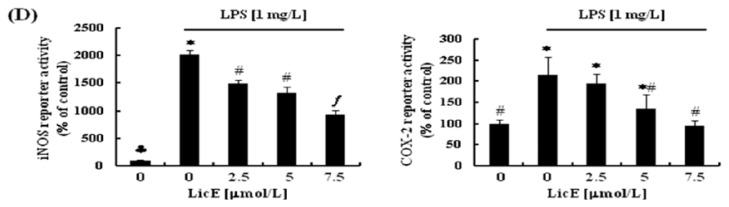
Licochalcone E (LicE) suppresses the production of NO and PGE_2_ as well as the expression of iNOS and COX-2 in LPS-stimulated RAW 264.7 cells. Serum-deprived RAW 264.7 cells were treated with various concentrations (0–7.5 μmol/L) of LicE in the absence or presence of 1 mg/L LPS for 24 h. (**A**) The 24 hour-conditioned media were collected for NO and PGE_2_ assays. Each bar represents the mean ± SEM from four independent experiments; (**B**) Total cell lysates were subjected to Western blotting with an anti-iNOS, COX-2, and β-actin antibody. The relative abundance of each band was quantified, and the LPS control levels (1 mg/L LPS + 0 μmol/L LicE) were set to 100%. ND, not detectable; (**C**) Total RNA was isolated and reverse transcribed, and real-time PCR was performed. The abundance of mRNA was quantified and the control levels (0 μmol/L LicE + 0 mg/L LPS) were set to 1. Each bar represents the mean ± SEM from three independent experiments; (**D**) RAW 264.7 cells were co-transfected with the murine iNOS or COX-2 reporter gene construct and renilla control vector, and the transfected cells were plated in 24-well plates at 5 × 10^4^ cells/well. After serum deprivation, the cells were treated for 6 h with the indicated concentrations of LicE in the presence of LPS. Cell lysates were prepared to measure luciferase activity. Luciferase activity was normalized to renilla activity. Each bar represents the mean ± SEM from three independent experiments. Means without a common symbol are statistically different (*p* < 0.05).

**Figure 4:**
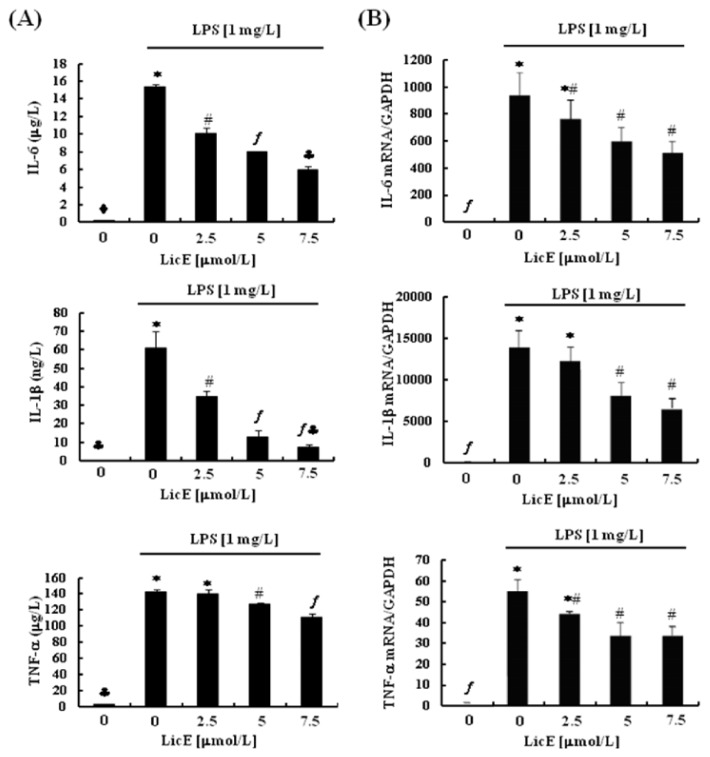
Licochalcone E (LicE) suppresses LPS-induced mRNA expression of IL-6, IL-1β, and TNF-α in RAW 264.7 cells. Serum-deprived RAW 264.7 cells were treated with various concentrations (0–7.5 μmol/L) of LicE in the absence or presence of 1 mg/L LPS for 24 h. (**A**) The 24 h-conditioned media were collected for ELISA. Each bar represents the mean ± SEM from four independent experiments; (**B**) Total RNA was isolated and reverse transcribed, and real-time PCR was performed. The abundance of mRNA was quantified and the control levels (0 μmol/L LicE + 0 mg/L LPS) were set to 1. Each bar represents the mean ± SEM from three independent experiments. Means without a common symbol are statistically different (*p* < 0.05).

In order to explore the detailed mechanisms by which LicE exerts anti-inflammatory properties we next examined whether LicE inhibits LPS-stimulated inflammatory responses in RAW 264.7 cells. LicE (2.5–7.5 μmol/L) dose-dependently inhibited LPS-induced secretion of NO and also drastically inhibited the secretion of PGE_2_ (Figure 3A). LicE markedly suppressed LPS-induced expression of iNOS and COX-2 proteins (Figure 3B) and the secretion of IL-6, IL-1β and TNF-α (Figure 4A). To examine whether LicE regulates these inflammatory mediators at an RNA levels, we estimated the mRNA levels of these cytokines and enzymes in RAW 264.7 murine macrophages by conducting real-time RT-PCR analyses. LPS markedly induced the mRNA levels of iNOS, COX-2, IL-6, IL-1β and TNF-α, which were significantly inhibited by LicE treatment (Figures 3C and 4B). Furthermore, LicE reduced the LPS-induced increases in iNOS and COX-2 promoter activities (Figure 3D). However, at the same concentrations, LicE did not influence the cell viability (data not shown).

### 2.3. LicE Suppresses LPS-Induced NF-κB Signaling in RAW 264.7 Cells

**Figure 5:**
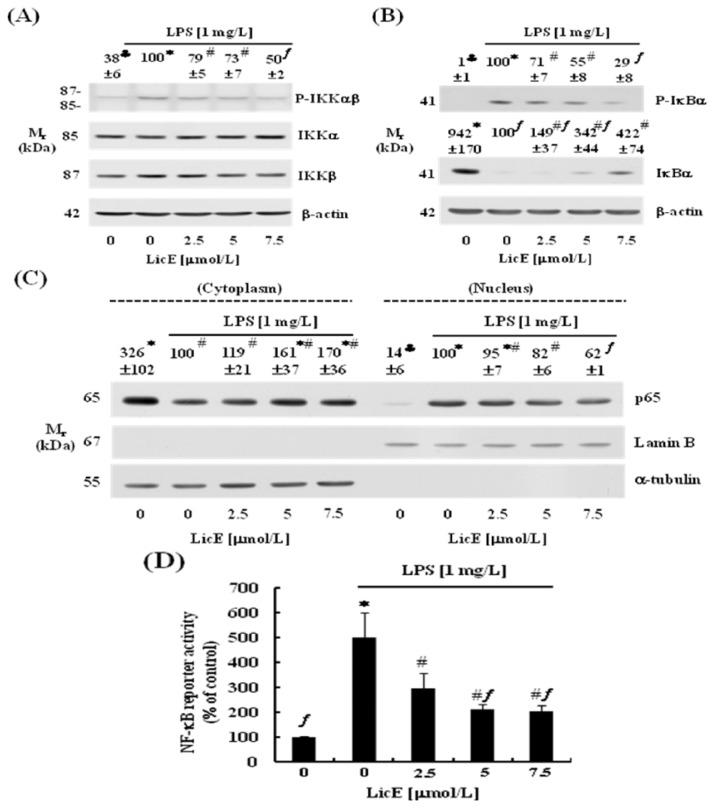
Licochalcone E (LicE) suppresses LPS-induced NF-κB signaling in RAW 264.7 cells. Serum-deprived cells were treated with various concentrations (0–7.5 μmol/L) of LicE for 40 min. LPS was then added and incubated for an additional 10 min (for the determination of P-IKKαβ, IKKα, IKKβ and P-IκBα) or 20 min (for the determination of IκBα and p65). (**A**,**B**) Total cell lysates were subjected to Western blotting with an anti-P-IKKαβ, IKKα, IKKβ, P-IκBα, IκBα or β-actin antibody; (**C**) Cytosolic and nuclear extracts were subjected to Western blotting with an anti-p65, Lamin B or α-tubulin antibody. The relative abundance of each band was quantified, and the LPS control levels (1 mg/L LPS + 0 μmol/L LicE) were set to 100%. The adjusted mean ± SEM (*n* = 3) of each band was provided above each blot; (**D**) RAW 264.7 cells were cotransfected with NF-κB reporter gene construct and renilla control vector, and the transfected cells were plated and treated as described in Figure 3D. Cell lysates were prepared to measure luciferase activity. Each bar represents the mean ± SEM from three independent experiments. Means without a common symbol are statistically different (*p* < 0.05).

We next determined whether LicE could inhibit LPS-stimulated activation of NF-κB signaling in RAW 264.7 cells. LPS-stimulated phosphorylation of IKK-αβ was significantly decreased by LicE treatment (Figure 5A). LicE also decreased LPS-induced phosphorylation of IκBα and degradation of IκBα (Figure 5B). Additionally, Western blot analysis of cytosolic and nuclear fractions showed that the LPS-induced translocation of NF-κB p65 to the nucleus was also reduced by LicE treatment (Figure 5C). Results from luciferase reporter gene assays revealed that LicE significantly inhibited LPS-induced transcriptional activity of NF-κB (Figure 5D).

### 2.4. LicE Suppresses LPS-Induced AP-1 Signaling

**Figure 6:**
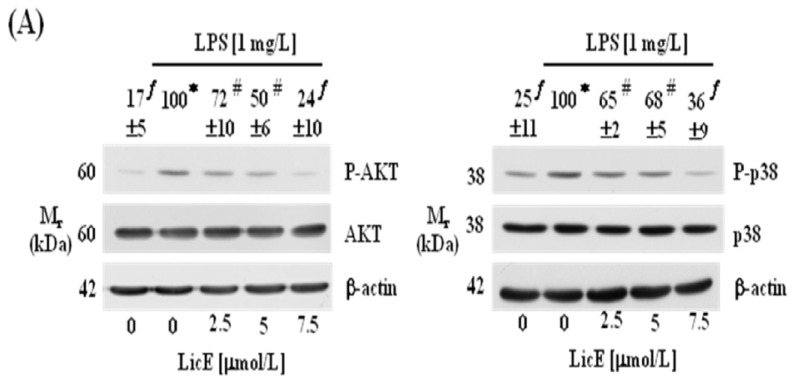

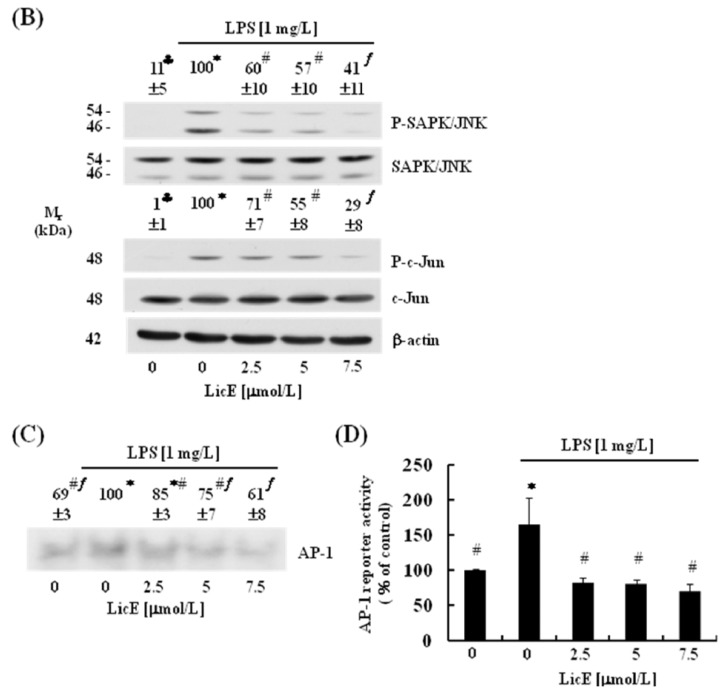
Licochalcone E (LicE) suppresses LPS-induced AKT, MAPK and AP-1 signaling in RAW 264.7 cells. (**A**–**C**) Serum-deprived RAW 264.7 cells were treated with LicE for 40 min. LPS was then added and incubated for an additional 20 min. (A,B) Total cell lysates were subjected to Western blotting with their relevant antibodies; (C) Nuclear extracts were prepared for electrophoretic mobility shift assay. Photographs of chemiluminescent detection of the blots (A,B) or an autoradiography of the dried gels (C) are shown. The relative abundance of the phosphorylated band to its own total protein (A,B) or each band (C) was quantified, and the LPS control levels (1 mg/L LPS + 0 μmol/L LicE) were set to 100%; (**D**) RAW 264.7 cells were cotransfected with AP-1 reporter gene construct and renilla control vector, and the transfected cells were plated and treated as described in Figure 3D. Cell lysates were prepared to measure luciferase activity. Each bar represents the mean ± SEM from three independent experiments. Means without a common symbol are statistically different (*p* < 0.05).

**Figure 7:**
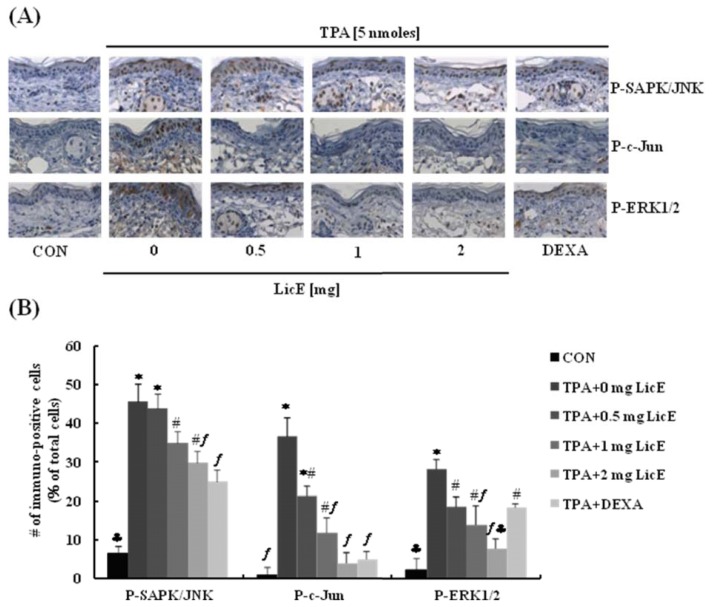
Licochalcone E (LicE) inhibits TPA-induced phosphorylation of SAPK/JNK, c-Jun and ERK1/2 in mouse skin. LicE (0, 0.5, 1, or 2 mg) was topically applied to the mouse ear for 1 h prior to the topical application of 5 nmoles of TPA (4 h). Ear sections were stained with an antibody raised against P-SAPK/JNK, P-c-Jun or P-ERK1/2 and counterstained with hematoxylin. (**A**) Representative images of the immunohistochemical analysis are shown; (**B**) P-SAPK/JNK, P-c-Jun and P-ERK1/2-positive cells were counted and expressed as a percentage of the total number of hematoxylin-stained cells. Each bar represents the mean ± SEM (*n* = 3). Means without a common symbol are statistically different (*p* < 0.05).

Because the activation of AP-1 leads to gene expression of inflammatory mediators, Western blot analyses for c-Jun and various upstream signaling proteins were conducted. In RAW 264.7 cells LicE treatment inhibited LPS-induced phosphorylation of AKT and p38 MAPK (Figure 6A). LicE also suppressed LPS-induced phosphorylation of SAPK/JNK and c-Jun, a downstream target of SAPK/JNK and a component of AP-1, in RAW 264.7 cells (Figure 6B). Furthermore, LicE treatment also inhibited LPS-induced DNA-binding activity (Figure 6C) and transcriptional activity (Figure 6D) of AP-1 in RAW 264.7 cells. Consistent with results observed in RAW 264.7 cell culture, LicE reduced TPA-induced expression of phosphorylated SAPK/JNK, c-Jun and ERK1/2 in mouse skin (Figure 7).

### 2.5. Discussion

Licorice root is one of the most frequently prescribed plant products in Oriental medicine and has been shown to exert anti-inflammatory properties [20]. LicE was recently isolated and characterized from the roots of *Glycyrrhiza inflate* [23]. Presently, we examined whether LicE exerts anti-inflammatory properties in TPA-stimulated mouse skin. Topical application of LicE (0.5–2 mg) markedly inhibited TPA-induced ear edema formation as well as the expression of iNOS and COX-2 proteins in mouse skin (Figure 2). LicE also effectively inhibited TPA-induced phosphorylation of ERK1/2, SAPK/JNK and c-Jun in mouse skin (Figure 7). These effects were almost comparable to those of dexamethasone, a widely used powerful anti-inflammatory agent.

In the present study, topical administration of LicE (0.5–2 mg) suppressed TPA-induced ear edema in mice. Currently, it is not possible to relate these doses to a human treatment. Cho and colleagues reported that the topical administration of LicE (20 μg–1 mg) reduced oxazolone-induced chronic allergic contact dermatitis [24]. Additionally, we recently reported that LicE (7–14 mg/kg body weight) inhibits solid tumor growth and lung metastasis of mammary carcinoma cells in a Balb/C mouse orthotopic model [25]. LicE is a lipophilic compound so that it can easily traverse through the hydrophobic region of the plasma membrane. Together, these results indicate that LicE is bioavailable to animals when it is administered topically or orally. However, future studies are needed to explore the mechanisms by which LicE is absorbed in the gut and distributed to target tissues as well as how it is penetrated into skin epithelial cells in humans.

In the present study we noted that the ability of LicE to inhibit TPA-induced skin inflammation is comparable to that of dexamethasone that is a synthetic glucocorticoid with potent anti-inflammatory activities. However, when dexamethasone is administered orally or parenterally more than a few days, a wide variety of adverse effects have been reported to occur [26]. Therefore, natural compounds which have a powerful anti-inflammatory activities with no or minimal side effect(s) are particularly useful to prevent inflammation. Actually, when licochalcone E (7–14 mg/kg of body weight) was orally administered in mice for more than 4 weeks, no kidney or liver toxicity was observed [25]. Future studies are warranted to explore the possibility of whether licochalcone E can be used as an anti-inflammatory agent in humans.

Macrophages actively participate in inflammatory responses by releasing a sequence of pro-inflammatory cytokines (IL-6, TNF-α and IL-1) and mediators (NO and PGE_2_) that recruit additional immune cells to the sites of inflammation. Thus, suppression of the release of these molecules is a good strategy to inhibit inflammation-related diseases [27–29]. In the present experiments, we noted that LicE decreases the LPS-induced protein expression of iNOS and COX-2, as well as the secretion of IL-6, IL-1β and TNF-α (Figures 3B and 4A). While this study was in progress, Park *et al.* reported *in vitro* preliminary results showing that LicE inhibited the generation of NO, PGE_2_, IL-6 and TNF-α, as well as the expression of iNOS and COX-2 proteins in RAW 264.7 cells [30]. Together, these results are consistent with the *in vivo* results that LicE exerts powerful anti-inflammatory properties.

In the present study, we have also demonstrated that LicE inhibited LPS-induced iNOS and COX-2 mRNA expression (Figure 3C). Additionally, the reporter activities of iNOS and COX-2 promoters were also reduced in LicE-treated RAW 264.7 cells (Figure 3D) indicating that LicE inhibits the production of NO and PGE_2_ by down-regulating the expression of *iNOS* and *COX-2* genes.

The NF-κB family of transcription factors performs a vital role in coordinating the expression of a broad variety of pro-inflammatory genes including iNOS, COX-2, IL-1, IL-6 and TNF [13]. Phosphorylation of IκBs by IKK marks them for polyubiquitination. Upon ubiquitination, the IκB proteins are degraded by the proteasome, thereby liberating NF-κB. NF-κB is then translocated to the nucleus where it binds to DNA and activates transcription (reviewed in [31]). We presently noted that LicE inhibited LPS-induced phosphorylation of IKK (Figure 5A) and IκBα (Figure 5B), degradation of IκBα (Figure 5B), translocation of NF-κB to the nucleus (Figure 5C) and NF-κB transcriptional activity (Figure 5D) in murine macrophages. These results indicate that inhibition of NF-κB signaling contributes to the reduced mRNA expression of iNOS, COX-2, IL-1β, IL-6 and TNFα (Figures 3C and 4B). However, it has been also reported that stimulation of HeLa cells with either TNFα or IL-1 increases the phosphorylation of IKKα and IKKβ [32] suggesting that reduced TNFα and/or IL-1β might have led to reduced IKK activation in LicE-treated macrophages. It remains to be determined the detailed mechanisms by which LicE inhibits the activation of IKK.

AP-1 is also a key transcription factor regulating the expression of iNOS and COX-2 which are central mediators of inflammation [16]. We noted that LicE inhibited LPS-stimulated phosphorylation of SAPK/JNK and c-Jun, a downstream target protein of SAPK/JNK and a component of AP-1 in RAW 264.7 cells (Figure 6B) and mouse skin (Figure 7). We also noted that LicE inhibited LPS-stimulated AP-1 binding to DNA, and transcriptional activity of AP-1 in RAW 264.7 cells (Figure 6C,D). Together, these results indicate that LicE inhibits AP-1 activation, which contributes to the reduced transcription of iNOS and COX-2 genes.

Phosphoinositol 3-kinase/AKT and MAPKs including JNK, ERK1/2 and p38 MAPK have been well characterized as potential signaling pathways regulating NF-κB activation [33–35]. AP-1 activation was also shown to be dependent upon phosphorylation by MAPKs [15]. Presently, LicE inhibited LPS-stimulated phosphorylation of AKT and p38 MAPK in RAW 264.7 cells (Figure 6A) and TPA-induced phosphorylation of SAPK/JNK and ERK-1/2 in mouse skin (Figure 7). The activation of MAPKs regulates TPA-induced expression of COX-2 in mouse skin [36]. Together, these results indicate that LicE inhibition of these kinases are involved in the inhibition of NF-κB and AP-1 activation, which leads to the reduced transcription of the inflammatory enzymes COX-2 and iNOS and the inflammatory cytokines IL-1β, IL-6 and TNF-α.

## 3. Experimental Section

### 3.1. Materials

Antibodies against iNOS and COX-2 were purchased from BD Transduction Laboratories (Palo Alto, CA, USA). Antibodies against inhibitor of κB kinase (IKK)β, NF-κB p65, lamin B and α-tubulin were purchased from Santa Cruz Biotechnology (Santa Cruz, CA, USA). Antibodies against P-IKKαβ (Ser-176/180), IKKα, IκBα, P-IκBα (Ser-32), AKT, P-AKT (Ser-473), stress-activated protein kinase/c-Jun-*N*-terminal kinase (SAPK/JNK), P-SAPK/JNK (Thr-183/Tyr-185), c-Jun, P-c-Jun (Ser-63), P-extracellular signal regulated kinase (ERK)-1/2 (Thr-202/Tyr-204), p38 mitogen activated protein kinase (MAPK) and P-p38 MAPK (Thr-180/Tyr-182) were purchased from Cell signaling (Beverly, MA, USA). LicE was a generous gift by Professor Jong-Gab Jun (Department of Chemistry, Hallym University, Chuncheon, Korea) [37]. Unless otherwise noted, all other materials were acquired from Sigma-Aldrich (St. Louis, MO, USA).

### 3.2. *In Vivo* Inflammation Model

All animal experimental protocols were approved by the Animal Care and Use Committee of Hallym University (Hallym2011-04). Four-week-old female ICR mice (Koatech, Seoul, Korea) were acclimatized to laboratory conditions for 1 week by providing laboratory chow and tap water *ad libitum*, and were then divided into six groups. TPA (5 nmol) and LicE (0.5–2 mg) were dissolved in 20 μL of dimethyl sulfoxide (DMSO)/acetone (3:17, *v:v*). Ear edema was induced by the topical application of 5 nmoles of TPA per ear. To assess the effects of LicE on ear edema formation, LicE (0.5–2 mg) or dexamethasone (DEXA, 50 μg) was topically applied to the ear 1 h prior to the TPA treatment. The control animals were treated with DMSO/acetone (vehicle). 4 h after the TPA treatment, the animals were sacrificed by carbon dioxide asphyxiation and the weights of the 6-mm diameter ear punch samples were individually measured.

### 3.3. Immunohistochemistry (IHC)

The ear samples were fixed in 40 g/L of paraformaldehyde for hematoxylin and eosin (H&E) staining and IHC. IHC analysis were performed as previously described [38]. Randomly chosen fields were photographed at 400× magnification, and ear thickness, immuno-positive cells were quantified with an AxioImager microscope and Image M1 Software (Carl Zeiss, Jena, Germany).

### 3.4. Cell Culture and Assays of Cell Viability, NO, PGE_2_ and Cytokines

RAW 264.7 macrophages (American Type Culture Collection, Manassas, VA, USA) were maintained in Dulbecco’s Modified Eagle Medium (DMEM) containing 100 mL/L fetal bovine serum (FBS), 100,000 U/L of penicillin and 100 mg/L of streptomycin. The effects of LicE (0–7.5 μmol/L) on cell viability were determined in the presence of 1 mg/L of lipopolysaccharide (LPS) by the 3-[4,5-dimethylthiazol-2-yl]-2,5-diphenyltetrazolium bromide (MTT) assay as described previously [39]. The concentrations of NO, PGE_2_ and cytokines (IL-1β, IL-6 and TNF-α) in media conditioned by RAW 264.7 cells for 24 h were estimated as previously described [39].

### 3.5. Western Blot Analysis

RAW 264.7 cells were plated in 100 mm-diameter dishes at 1 × 10^6^ cells per dish with DMEM containing 100 mL/L FBS, serum deprived in DMEM containing 10 mL/L FBS and treated with various concentrations (0–7.5 μmol/L) of LicE in the absence or presence of 1 mg/L LPS. Cell lysates and nuclear extracts were prepared and Western blot analyses were conducted as previously described [39]. Signals were detected by enhanced chemiluminescence using Immobilon™ Western Chemiluminescent HRP Substrate (Millipore, Billerica, MA, USA). Densitometric analysis was conducted using the Bioprofile BioID application (Vilber Lourmat, France). Expression levels were normalized to β-actin, α-tubulin or lamin B and the LPS control (1 mg/L LPS + 0 μmol/L LicE) levels were set at 100%.

### 3.6. Real-Time RT-PCR

Total RNA was isolated using RNeasy Plus Mini Kit (Qiagen, Valencia, CA, USA) and cDNA was synthesized as described previously [39]. For quantification of iNOS, COX-2, IL-6, IL-1β, TNF-α and glyceraldehyde-3-phosphate dehydrogenase (GAPDH) transcripts, real-time PCR was performed using a Rotor-gene 3000 PCR (Corbett Research, Mortlake, NSW, Australia) as described previously [39]. The analysis of PCR results and calculation of the relative concentrations were performed using the Rotor-gene software (version 6) and the control levels (0 μmol/L LicE + 0 mg/L LPS) were set at 1. Sequences for PCR primer sets have been previously published for IL-6, IL-1β, TNF-α and GAPDH [40] and for iNOS and COX-2 [41].

### 3.7. Luciferase Reporter Gene Assay

RAW 264.7 cells were co-transfected with pGL-miNOS-1588 [42], pGL-mCOX-2-724 [43], NF-κB-luciferase reporter plasmid (Takara Bio, Otsu, Shiga, Japan) or pAP-1-luciferase (Takara Bio) reporter plasmid [44] and pRL-TK renilla control vector (Promega, Madison, WI, USA), using Nucleofector-II (Amaxa, Gaithersburg, MD, USA). The transfected cells were plated in 24-well plates at 5 × 10^4^ cells/well. After 24 h, cells were serum-deprived for 24 h, and then incubated for 6 h with 0–7.5 μmol/L of LicE in the absence or presence of LPS. Cell lysates were prepared to measure luciferase activity using the Dual Luciferase Assay System (Promega) according to the manufacturer’s instructions.

### 3.8. Electrophoretic Mobility Shift Assay (EMSA)

Nuclear extract was prepared and EMSA was conducted as previously described [39]. Double-stranded DNA probe for the consensus sequence of AP-1 (5′-CGC TTG ATG AGT CAG CCG GAA-3′) was used for EMSA after end-labeling of probe with [γ-^32^P] ATP and T4 kinase. Each sample was subjected to 5% nondenaturing gel, and the gels were dried and visualized via autoradiography.

### 3.9. Statistical Analyses

Data are expressed as mean ± SEM and were analyzed by ANOVA. Differences between treatment groups were analyzed by Duncan’s multiple range test, using SAS for Windows, version 9.1 (SAS Institute). A *p* value < 0.05 was considered statistically significant.

## 4. Conclusions

In summary, we have demonstrated that LicE isolated from licorice exhibits potent anti-inflammatory properties in mouse skin and murine macrophages. LicE inhibited LPS-induced iNOS and COX-2 mRNA expression as well as their promoter activities. LicE also inhibited the secretion of the pro-inflammatory cytokines IL-1β, IL-6 and TNF-α and their corresponding mRNAs. Furthermore, LicE inhibited NF-κB and AP-1 signaling and phosphorylation of AKT, SAPK/JNK, p38 MAPK and ERK1/2. Inhibition of AKT and MAPK activation by LicE may contribute to the inhibition of AP-1 and NF-κB signaling, which, in turn, leads to decreased expression of inflammatory cytokines and inducible enzymes.
